# The Comparison of the Selected Parameters of Brain Injury and Interleukins in the CSF in Patients Diagnosed De Novo with RRMS Compared to the Control Group

**DOI:** 10.3390/diagnostics13223436

**Published:** 2023-11-13

**Authors:** Bożena Adamczyk, Natalia Morawiec, Gabriela Mamak, Sylwia Boczek, Dominika Brzęk, Natalia Trędota, Patryk Walocha, Zenon P. Czuba, Michał Błachut, Wojciech Bartman, Monika Adamczyk-Sowa

**Affiliations:** 1 Department of Neurology, Faculty of Medical Sciences in Zabrze, Medical University of Silesia in Katowice, ul. 3 Maja 13-15, 41-800 Zabrze, Poland; nataliamorawiec007@gmail.com (N.M.); mamak.gabriela@gmail.com (G.M.); s79050@365.sum.edu.pl (S.B.); s78413@365.sum.edu.pl (D.B.); naiilaan@gmail.com (N.T.); s81574@365.sum.edu.pl (P.W.); wbartman@sum.edu.pl (W.B.); m.adamczyk.sowa@gmail.com (M.A.-S.); 2 Department of Microbiology and Immunology, Faculty of Medical Sciences in Zabrze, Medical University of Silesia in Katowice, ul. Jordana 19, 41-808 Zabrze, Poland; zczuba@sum.edu.pl; 3 Clinical Department of Psychiatry, Faculty of Medical Sciences in Zabrze, Medical University of Silesia in Katowice, 40-055 Katowice, Poland; mblachut@sum.edu.pl

**Keywords:** multiple sclerosis, NF-H, GFAP, S100B, UCHL1, interleukins

## Abstract

Background: Multiple sclerosis (MS) is a chronic autoimmune disorder affecting the central nervous system (CNS). Due to the different phenotypes of the disease and non-specific symptoms of MS, there is a great need for a validated panel of biomarkers to facilitate the diagnosis, predict disease progression, and evaluate treatment outcomes. Methods: We determined the levels of the parameters of brain injury (NF-H, GPAF, S100B, and UCHL1) and the selected cytokines in the cerebrospinal fluid (CSF) in 101 patients diagnosed de novo with RRMS and 75 healthy controls. All determinations were made using the Bio-Plex method. Results: We found higher levels of NF-H and GFAP in the relapsing-remitting multiple sclerosis (RRMS) group compared to the controls. The concentrations of both molecules were significantly increased in patients with Gd+ lesions on brain MRI. The level of S100B did not differ significantly between the groups. UCHL1 concentrations were higher in the control group. We found some correlations between the selected cytokines, the levels of the parameters of brain injury, and the time from the first symptoms to the diagnosis of MS. Conclusions: The role of the above molecules in MS is promising. However, further research is warranted to define their precise functions.

## 1. Introduction

Multiple sclerosis (MS) is a chronic inflammatory disorder of the central nervous system (CNS) caused by an autoimmune attack on the myelin sheath and the cells that produce and maintain it. This process leads to neurodegeneration in the brain and spinal cord. MS typically affects young adults, causing various neurological symptoms [1,2]. Exogenous, environmental, and genetic factors may be involved in the development of the disease. However, the etiology is not fully elucidated [3].

There are four phenotypes of MS, depending on their clinical course: relapsing-remitting multiple sclerosis (RRMS), primary progressive multiple sclerosis (PPMS), secondary progressive multiple sclerosis (SPMS), and clinically isolated syndrome (CIS) [3]. Inflammation is prevalent in all phenotypes of MS [4]. RRMS is the most common phenotype found in 85% of patients with MS. The relapse rate is estimated to be between 0.4 and 1.2 per year. Remission occurs between relapses. At the onset of the disease, remissions are almost complete. However, relapses gradually lead to disability as the disease progresses [3].

Demyelinated areas with destruction and loss of oligodendrocytes are the most typical features of MS [2]. Even when myelin is entirely gone, axons are retained to varying degrees. The inflammatory activation of lymphocytes B, T, and plasma cells causes pathological alterations. Demyelination activates astrocytes, resulting in the formation of gliotic scars [2]. Because of the activation of oligodendrocyte progenitor cells, partial remyelination is also possible [2].

Inflammatory mechanisms cause demyelination and neurodegeneration. Current research indicates that neurodegeneration may trigger MS [2]. It has been hypothesized that inflammation modifies the primary process of neurodegeneration [5]. MS is distinguished by neuronal and axonal loss, astrocytic gliosis, and demyelination. Axonal loss can occur suddenly, due to new inflammatory events, or gradually in chronic demyelination [6]. Axonal loss mechanisms have been documented, including neuronal energy deficit, myelin trophic support loss, and the production of reactive oxygen species (ROS) or nitric oxide (NO) [7,8]. ROS and NO can generate mitochondrial dysfunction. That may result in the variety of degenerative characteristics seen in MS [7,8]. Mitochondrial dysfunction can also contribute to ATP generation and higher calcium levels, which can lead to neuronal death [7,9].

Due to chronic inflammation, an imbalance between damage, repair, and functional reserve of the brain is observed [4]. Progressive neuronal damage is reflected by the increased levels of biomarkers of brain injury. They are of great value in assessing the stage of the disease, the prognosis, and the response to treatment. Various potential parameters of brain injury, such as NF-H, GFAP, S100B, and UCHL1, have been described in the literature. However, there is still limited data on their function in MS [10].

Neurofilaments (NFs) are the elements of the neuronal cytoskeleton that are particularly abundant in axons. They provide structural support and maintain the size and shape of the axons. NFs belong to the family of intermediate filaments and are composed of three subunits: light-chain neurofilament (NF-L), medium neurofilament (NF-M), and heavy neurofilament (NF-H) [11]. Following axonal damage in the CNS, neurofilament proteins are released into the cerebrospinal fluid (CSF), which indicates axonal damage and neuronal death. NF-L is the most extensively studied subtype in this context [12,13]. In RRMS, neurofilaments are associated with the clinical and radiological activity of the disease and may predict disability progression [14]. Neurofilaments as biomarkers have been the subject of intensive research since the discovery of elevated NF-L levels in patients with RRMS [13].

Glial fibrillary acidic protein (GFAP) is a type III intermediate filament protein that belongs to the family of cytoskeletal proteins. It is expressed in many types of CNS cells, primarily in the cytoplasm of astrocytes. GFAP ensures astrocyte stability, maintains astrocyte shape, and is a marker for astroglial activation [15]. GFAP expression is usually low in resting astrocytes. GFAP secretion is increased in response to brain injury and astrocyte hypertrophy. This reaction is known as reactive gliosis, in which astrocyte cells undergo functional changes in response to CNS injury. Neuroinflammation has been connected to the pathophysiology of many neurological conditions and neurochemical biomarkers, including GFAP, which may be useful biofluid markers of brain injury correlated with neurodegeneration [15,16,17,18,19,20]. Studies have shown that GFAP levels are elevated in MS patients. High GFAP levels are correlated with increased disease activity, disability progression, and pathology on MRI. For this reason, GFAP has gained interest as a potential biomarker for assessing MS severity, monitoring response to treatment, and predicting disease progression [17,21,22,23].

S100B is a helix-loop-helix protein with a calcium-binding domain [24,25]. By increasing nuclear factor kappa B (NFkB) expression and activating the mitogen-activated protein kinase (MAPK) pathway, this molecule prolongs cell life and promotes cell proliferation [25]. Astrocytes are the immediate target of the protein. S100B also increases nitric oxide (NO) levels and causes neuroinflammation, neurodegeneration, and impairment of axonal conduction [25,26,27]. Therefore, S100B protein levels are increased in many neurological diseases, including MS, Alzheimer’s disease, Parkinson’s disease, schizophrenia, and epilepsy [25].

Another parameter of brain injury is ubiquitin C-terminal hydrolase-L1 (UCHL1), which is a proteolytically stable small protein of neuronal origin that participates in repairing injured axons and immunological reactions. Studies showed that the levels of this protein were increased in serum and CSF in patients after traumatic brain injury and were linked with the intensity of injury and long-term outcome [28,29,30,31]. The enzyme is expressed at high concentrations in neurons of the brain and spinal cord and plays a significant role in the normal functioning of the nervous system [32].

As previously noted, all kinds of MS are characterized by neuroinflammation, and mediators, such as cytokines, play an essential role in the pathophysiology of the disease [4]. Interleukins (ILs) belong to the cytokine family. They are produced by cells such as macrophages, eosinophils, vascular endothelial cells, fibroblasts, and keratinocytes. These chemicals influence inflammatory and immunological processes, as well as cell development and differentiation [33,34,35]. Proinflammatory cytokines participate in neuroinflammation and contribute to the development of MS, while some anti-inflammatory mediators, which are secreted in smaller amounts, may have a protective effect. The mechanisms of IL signal transduction seem to be evident. However, their action is not entirely described [36].

The aim of this study was to compare the levels of the above parameters of brain injury between patients diagnosed de novo with RRMS and a group of healthy controls without demyelinating changes in the brain. Additionally, we assessed whether there was a correlation between the levels of NF-H, GFAP, S100B, and UCHL1 and the radiological activity of the disease. We checked whether there were any significant correlations between brain injury markers and the selected proinflammatory and anti-inflammatory cytokines. This study addressed an important topic as it evaluated the usefulness of the four new markers in the diagnostic process of RRMS, whose symptoms in the early stages of the disease may only manifest themselves as demyelinating changes in the brain.

## 2. Materials and Methods

We enrolled 101 patients diagnosed de novo with RRMS and 75 healthy controls. Among these participants, we mainly observed clinical symptoms such as vision disorders, vertigo, balance disorders, weakness of limbs, sensory disturbances (paresthesia, numbness, tingling), and optic neuritis, which were the reasons to start the diagnostic process to confirm MS. In the group of healthy controls, demyelination was excluded. These patients mostly complained about headaches and vertigo, which in this case prompted us to perform an extended diagnostic evaluation. The cerebrospinal fluid (CSF) was collected during the diagnostic process to confirm or rule out MS. De novo diagnosed RRMS patients underwent MRI of the brain, cervical, and thoracic spine and were assessed in terms of the number of T2-weighted lesions and Gd+ lesions. In further analysis of the MRI results, we focused mainly on gadolinium-enhancing lesions. This allowed us to detail the correlations between Gd+ lesions and the studied molecules, which are crucial in the development of de novo MS. The participants met the following enrollment criteria: age > 18 years, RRMS diagnosed de novo according to the 2017 McDonald criteria [37], and no prior disease-modifying therapy. The exclusion criteria were as follows: no written informed consent, another neurological condition, or a serious illness that could significantly affect the examination results. Moreover, patients experiencing disease relapse and steroid therapy were not included in the study. The study was approved by the Bioethics Committee of the Medical University of Silesia in Katowice (consent no. KNW/0022/KB1/37/16).

The concentrations of the parameters of brain injury, such as NF-H, GFAP, S100B, and UCHL1, were determined (Invitrogen 4-Plex Brain Injury, Carlsbad, CA, USA). We also assessed the levels of the selected proinflammatory and anti-inflammatory cytokines (IL-1β, IL-1RA, IL-2, IL-4, IL-5, IL-6, IL-7, IL-8, IL-9, IL-10, IL-12, IL-13, IL-15, IL-17, TNF-α, and IFN-γ) using the Bio-Plex Pro Human Cytokine 27-plex Assay. The determinations were performed according to the manufacturer’s instructions. The Bio-Plex method with the Bio-Plex 200 apparatus (Bio-Rad, Hercules, CA, USA) was applied [38].

Microsoft Excel (Microsoft^®^ Excel^®^ 2019 MSO (wersja 2309 kompilacji 16.0.16827.20166) 64-bitowa) was used to prepare the database for calculations. Statistical analysis was performed using Statistica Data Miner 14.0. *p* < 0.05 was considered statistically significant. We performed many comparisons between the levels of the selected factors in the study group and the controls. We also assessed the correlations between the related parameters. The compliance of the variables with a normal distribution was checked using the Shapiro–Wilk test. The *t*-test and the Mann–Whitney U test were used to compare the groups. Spearman’s linear correlation was used to assess the relationship between the individual variables. The ANOVA test and non-parametric Kruskal–Wallis ANOVA were used to assess the homogeneity of continuous variables between the groups.

## 3. Results

Women (72.27%) were predominant among the subjects. The median age at diagnosis of MS was 38.44 years, the median time from the first symptoms to diagnosis was 63.53 months, and the median number of T2-weighted lesions on brain MRI for the RRMS group was 16.72 (Table 1).

### 3.1. The Comparison of the Selected Parameters of Brain Injury and the Selected Interleukins in the CSF in Patients Diagnosed De Novo with RRMS and the Controls

**Figure 1:**
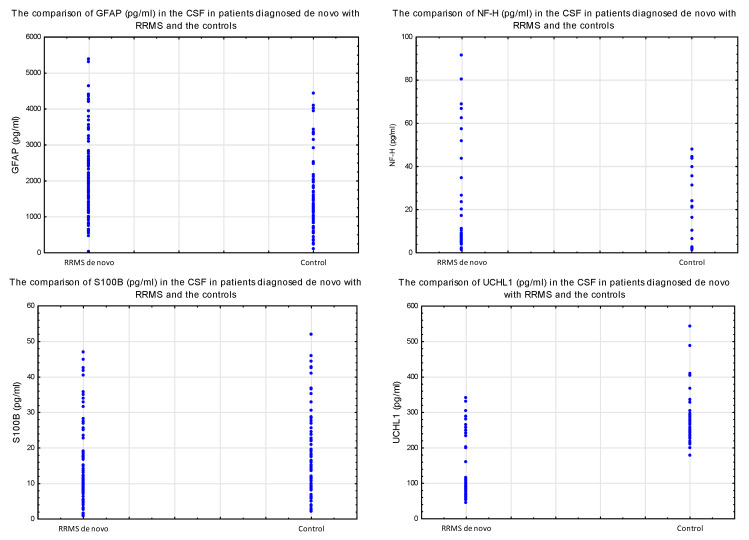
The comparison of the selected parameters of brain injury in the CSF in patients diagnosed de novo with RRMS and the control group.

The concentrations of GFAP and NF-H in the CSF were higher in the study group compared to the control group, while the concentration of UCHL1 was lower in the study group. The concentration of S100B did not differ between the groups (Table 2) (Figure 1).

#### 3.1.1. Correlations of the Selected Interleukins with the Selected Parameters of Brain Injury in the CSF in Patients Diagnosed De Novo with RRMS

In patients diagnosed de novo with RRMS, the concentration of GFAP correlated positively with the concentration of IL-8. The level of NF-H in the CSF increased with the concentrations of TNF-α, IL-7, and IL-10. UCHL1 correlated positively with IL-2 and IL-3. No correlations were noted for the S100B protein (Table 3).

#### 3.1.2. Correlations of the Selected Interleukins with the Selected Parameters of Brain Injury in the CSF in the Control Group

In the control group, the concentration of NF-H correlated positively with the concentrations of IL-6 and IL-8. The level of S100B in the CSF increased with the concentrations of IFN-γ, IL-5, IL-6, IL-7, IL-8, and IL-9. No correlations were noted for GFAP or UCHL1 (Table 3).

### 3.2. Correlations of the Selected Parameters of Brain Injury with the Selected Interleukins in the CSF in Patients Diagnosed De Novo with RRMS Depending on MRI Lesions and the Time from the First Symptoms to Diagnosis

**Figure 2:**
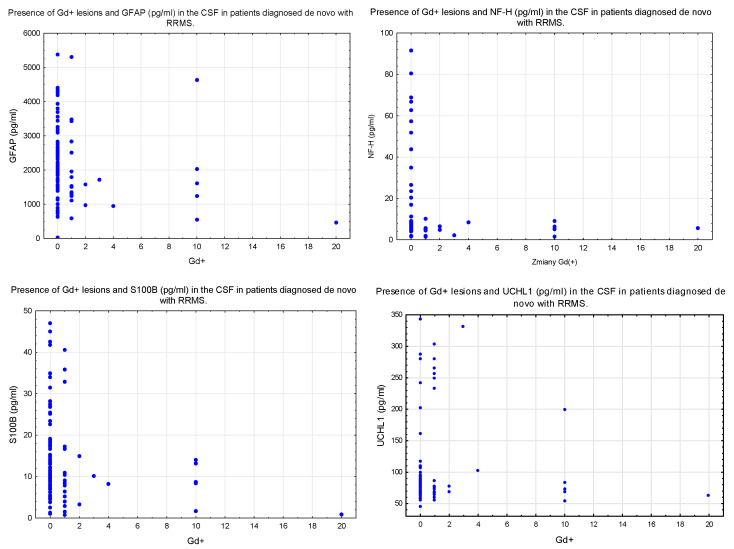
Presence of Gd+ lesions and the selected parameters of brain injury in the CSF in patients diagnosed de novo with RRMS.

The concentrations of GFAP and NF-H in the CSF were higher in the group with gadolinium-enhancing (Gd+) lesions on MRI compared to the group with no Gd+ lesions on MRI (Table 4 and Table 5) (Figure 2).

Time from the first symptoms to diagnosis correlated positively with the concentrations of IFN-γ and TNF-α, IL-1, IL-4, IL-5, IL-9, and IL-10. No correlations were noted for GFAP, NF-H, S100B, or UCHL1.

## 4. Discussion

### 4.1. Heavy Neurofilament (NF-H)

Heavy neurofilament (NF-H) is a promising biomarker of disease activity in RRMS. In our study, NF-H concentrations in the CSF were significantly higher in patients diagnosed de novo with RRMS than in healthy controls. Herrera et al. also showed increased NF-H concentrations in patients with 5 year RRMS evolution. In their study, a correlation between NF-H levels and disease progression was observed, which suggested its potential reliability in monitoring the clinical condition of RRMS patients [39]. Shehab et al. obtained similar results. Additionally, in their study, the pNF-H levels were significantly higher in the relapsing group than in the remitting group [40]. Another study revealed higher levels of pNF-H in the CSF of CIS patients that converted to RRMS in 3 years than in CIS patients who did not develop MS at the same time [41].

We found higher levels of NF-H in patients with Gd+ lesions on MRI. By combining the MRI findings of Gd+ lesions with the measurements of NF-H, clinicians can obtain a more comprehensive assessment of RRMS patients. The presence of Gd+ lesions may indicate the need for more aggressive treatment to control active inflammation and prevent further neurodegeneration. Elevated levels of NF-H may offer information to healthcare providers on the extent of neuroaxonal damage. As a result, different therapeutic choices can be applied with further prognostic evaluation [42].

Our study showed a correlation between NF-H levels and the concentrations of the selected interleukins. In our study group, a positive correlation was observed between NF-H levels and TNF-α, IL-7, and IL-10. In their study on the presence of NF-H and soluble TNF receptor 1 that reflects the biological activity of TNF-α in subacute sclerosing panencephalitis, Matsushige et al. found a significant correlation between NF-H and sTNFR1 levels in the CSF of SSPE patients. Further investigation is warranted to explore the potential relationship between TNF-α and NF-H levels as indicators of axonal damage and the progression of neurodegenerative diseases [43].

In the control group, the concentration of NF-H correlated positively with the concentrations of IL-6 and IL-8. The literature on the relationships between the levels of NF-H and the above interleukins in RRMS is very limited. The study attempted to determine selected correlations, but not all correlations in our study would be strong enough. This indicates that this topic requires further analysis in a larger group of patients. However, an interesting relationship was reported by Daoud et al. in their study on brain injury biomarkers as outcome predictors in pediatric severe traumatic brain injury (sTBI). The study indicated that elevated levels of IL-8 and NF-H correlated with unfavorable outcomes in sTBI patients, while elevated levels of nerve growth factor (NGF), doublecortin (DCX), and IL-6 correlated with favorable outcomes [44].

The above studies highlighted the importance of NF-H as a biomarker in many neurological conditions, including RRMS. NF-H has demonstrated promising diagnostic and prognostic potential and could serve as an indicator of disease progression and treatment effectiveness. Further research is warranted to fully understand the significance of NF-H in different neurological disorders and its correlation with interleukins, which may provide valuable insights for future therapeutic approaches.

### 4.2. Glial Fibrillary Acidic Protein (GFAP)

Glial fibrillary acid protein (GFAP) is another biomarker of neuroinflammation and a potential prognostic factor for MS severity. In RRMS patients, it is a promising indicator of astrogliosis and astroglial damage [23].

Our study found higher levels of GFAP in patients diagnosed de novo with RRMS than in the control group. GFAP correlated positively with the concentration of IL-8 in the study group. Moreover, we found an association between GFAP and Gd+ lesions on brain MRI.

Azzolini et al. showed that higher levels of GFAP in the CSF of newly diagnosed patients with RRMS were associated with an increased risk of disease progression. Similar to our study, Azzolini et al. found a significant correlation between GFAP levels and IL-8 [17]. IL-8 is a proinflammatory cytokine that is produced by astrocytes and microglia in response to inflammatory stimuli. Concentrations of IL-8 were higher in untreated patients with MS [45], and the levels of IL-8 in MS were associated with disease activity and disability [46,47].

Another study conducted by Kassubek et al. suggested that GFAP was a useful marker of RRMS. They found elevated levels of GFAP in the CSF of patients with early RRMS. Furthermore, they also observed a significant correlation between GFAP levels and Gd+ lesions on MRI, which indicated that the increased concentration of the molecule could be a relevant indicator of acute neuroinflammation [48]. In their meta-analysis, Momtazmanesh et al. showed that higher levels of GFAP were present in patients with PPMS compared to RRMS subjects [23]. This allows us to verify whether GFAP might be a good parameter to distinguish between the phenotypes of the disease in the early stages [49].

Based on our study results and the literature findings, GFAP seems to be a useful parameter in the early stage of MS by reflecting astrocyte activation and damage caused by immune-mediated inflammatory processes. Understanding these molecular mechanisms may help researchers develop better diagnostic tools and potential therapies for RRMS and other neuroinflammatory disorders.

### 4.3. S100B

S100B is an acidic homodimer synthesized mainly by astrocytes and a small subset of oligodendrocytes. It shows concentration-dependent intracellular and extracellular effects, exerting neurotrophic effects at nanomolar concentrations and neurotoxic effects at micromolar concentrations [24,25,50]. In the acute phase of MS, protein concentration increases. During periods of remission, its concentration decreases [50,51].

Our study found no significant changes in CSF S100B protein levels in patients diagnosed de novo with RRMS compared to controls. This finding is not in line with the results of earlier studies that showed increased S100B levels in the CSF of patients with MS [24,52,53]. These discrepancies in study results can be explained by the selection of the population. In these studies, the study population was different and included patients with other clinical phenotypes of MS or those with different stages of the disease in whom demyelination may have been more advanced. On the other hand, Barateiro et al. found increased S100B levels at the time of MS diagnosis but considered different phenotypes [54]. Further research on the course and various phenotypes of MS is necessary to explain the aforementioned inconsistencies and may help determine the role and use of S100B. In this study, we also evaluated the correlation between the concentration of the S100B molecule and the selected interleukins to evaluate the presence of specific dependencies relevant to the development of MS. No correlation was observed between increased S100B concentrations and interleukin levels in the study group. Different results were obtained by Santos et al. In their study, increased expression of S100B molecules correlated with the activity of TNF-α and IL-1β, promoting the formation of an inflammatory phenotype in neural tissue, which resulted from the involvement of RAGE receptors and stimulation of the activity of the transcription factor NF-κB [24]. The positive correlation between S100B levels and pro-inflammatory factors, such as TNF-α, IL-1β, or IL-6, was also reported by other authors, which proved the proinflammatory effect of elevated S100B levels [55,56].

To contrast the results of the study group and more accurately estimate the correlation of the S100B factor and interleukins, a similar evaluation was carried out in a control sample, which showed the existence of a correlation between the study protein and selected interleukins, as shown in Table 3. To accurately clarify and confirm the role played by the S100B protein, further studies, including a larger study group as well as a control group, are necessary.

Our study showed a positive correlation between interleukins and S100B in the controls. However, it was related to IFN-γ, IL-5, IL-6, IL-7, IL-8, and IL-9. A precise determination of the mechanism of action of S100B in MS requires further studies.

S100B is a widely accepted marker of neuronal damage. As a result, S100B leakage into the extracellular fluids is observed, resulting in its increased concentration [51].

We also examined the correlations between S100B levels and Gd+ lesions in the CSF of patients. We divided subjects into two groups, i.e., those with Gd+ lesions and those with no Gd+ lesions on MRI. Our study showed no increase in S100B levels in the group with Gd+ lesions. The evaluation and comparison of the above results is complex, as there are no other direct studies assessing the correlation between Gd+ lesions and S100B levels. Furthermore, only a few studies have described changes in S100B concentrations in relation to changes on MRI in other neurological conditions. In 2019, Gunawan et al. found a positive correlation between radiological changes and S100B concentration. However, their study was based on the assessment of children with epilepsy [57].

### 4.4. Ubiquitin C-Terminal Hydrolase-L1 (UCHL1)

UCHL1 is a deubiquitinating enzyme of neuronal origin whose role in vivo remains partially unknown. However, its high content in neurons in the brain and spinal cord suggests that it plays a significant part in the normal activity of the nervous system [32,58]. The proteolytically stable protein seems to be involved in restoring the appropriate function of damaged axons and neurons [32].

Moreover, it participates in the immune response, which might explain why the concentration of UCHL1 in patients diagnosed de novo with RRMS in our study correlated positively with the concentration of IL-2, known as a proinflammatory cytokine, and correlated positively with the concentration of IL-3, which is considered a multifunctional regulator of inflammation affecting immune cells and hematopoiesis [32,36,59].

To date, studies on the impact of UCHL1 on the course of MS have been very limited; hence, further investigation is warranted. Nevertheless, according to Górska et al., plasma UCHL1 could be a high-quality biomarker of RRMS. It was found that the plasma concentration of the enzyme was higher in RRMS patients compared to the control group, and it showed the highest diagnostic sensitivity (100%) compared to other markers in distinguishing MS patients from non-MS participants [32].

Our study showed higher concentrations in the control group than in the study group. The discrepancies between the results of UCHL1 levels in MS patients and the control group between the present study and the above research may be due to the assessment of the quantity of the enzyme in different body fluids, diverse research methodologies, and different characteristics of the cohorts. In this study, patients were diagnosed de novo with RRMS as opposed to the subjects in the study of Górska et al., which could have influenced the protein concentration.

### 4.5. Interleukins

Cytokines are involved in the immune response and may influence the disease course in MS. These small proteins act as signaling molecules in the immune system, regulating the immune response and promoting inflammation or anti-inflammatory effects. In MS, the imbalance between proinflammatory and anti-inflammatory cytokines can influence the severity and progression of the disease [60]. Our study found a correlation between the time from the first symptoms to the diagnosis of MS and the levels of IFN-γ, TNF-α, IL-1, IL-4, IL-5, IL-9, and IL-10. Some cytokines, such as IFN-γ and TNF-α, are proinflammatory and can contribute to the immune attack on myelin in the CNS. On the other hand, cytokines, including IL-4, IL-5, IL-9, and IL-10, have a more potent anti-inflammatory role and may help regulate the immune response and limit tissue damage [36]. As the inflammatory imbalance is seen in each phenotype of MS, our findings seem to be consistent with this statement. The longer the time from the first symptoms to diagnosis, the more intense the inflammatory process and dysregulation in the cytokine system.

However, our study has potential limitations. The first is the long observation period and the influence of the long sample storage on biomarker stability. Secondly, there is limited data on the brain injury markers among the patients with de novo-diagnosed RRMS. Due to the literature gaps, there is a need for further development in this area.

## 5. Conclusions

In conclusion, NF-H, GFAP, S100B, and UCHL1 are promising biomarkers that have been extensively studied for their potential roles in the prognosis and monitoring of RRMS. Our findings suggest that NF-H and GFAP, in particular, are useful markers of neuroinflammation in the early stage of RRMS. We found higher levels of these molecules in the CSF of patients diagnosed de novo with RRMS compared to healthy controls. Moreover, we showed an association between both parameters and Gd+ lesions on brain MRI. S100B did not significantly differ in both groups. UCHL1 concentrations were higher in the control group. Due to the limited data in the literature, further analysis is warranted to determine the precise role and significance of S100B and UCHL1 in MS.

Although the roles of NF-H, GFAP, S100B, and UCHL1 in RRMS are promising, further research is needed to fully understand their specific contributions and potential clinical utility. Standardization of measurement techniques, validation in larger and more diverse patient populations, and longitudinal studies are essential for their successful translation into clinical practice. Due to the long observation period in the study, the value of the assessed molecules in the CSF as diagnostic biomarkers in the de novo diagnosed RRMS patients may be limited.

However, there is a great need for a validated panel of biomarkers in MS that could allow the determination of the prognosis and response to treatment so that the most personalized immunomodulatory therapy could be introduced [61,62]. Integrating these biomarkers into the diagnostic and prognostic strategies for RRMS could improve early detection, treatment monitoring, and personalized therapeutic interventions, thus enhancing the management and outcomes for patients with this complex neurological disorder.

## Figures and Tables

**Table 1:** General characteristics of the groups.

	Study Group	Control
*N*	101	75
Gender (% of females)	72.27%	85.33%
Age * (years)	38.44 ± 11.78	38.88 ± 11.59
Median number of T2-weighted lesions on brain MRI	16.72	NA
Time from the first symptoms to the diagnosis (months)	63.53	NA

* In the study group, age was consistent with the age at diagnosis of MS. NA—Not applicable.

**Table 2:** The comparison of the selected parameters of brain injury in the CSF in patients diagnosed de novo with RRMS and the controls.

Parameter	RRMS Group	Control	*p*
*N*	101	75	
GFAP (pg/mL)	2208.91 ± 1137.16	1530.84 ± 992.57	0.001
NF-H (pg/mL)	12.15 ± 17.61	8.89 ± 12.14	0.001
S100B (pg/mL)	13.86 ± 10.72	16.79 ± 12.13	0.097
UCHL1 (pg/mL)	103.08 ± 68.48	272.03 ± 68.39	0.001

NF-H—neurofilament heavy chains, GFAP—glial fibrillary acidic protein, S100B—calcium-binding protein B, UCHL-1—ubiquitin C-terminal hydrolase L1, RRMS—relapsing-remitting multiple sclerosis.

**Table 3:** Correlations of the selected interleukins with the selected parameters of brain injury in the CSF in patients diagnosed de novo with RRMS and the controls.

	Study Group						Control					
Parameter	TNF-α	IL-2	IL-3	IL-7	IL-8	IL-10	IFN-γ	IL-5	IL-6	IL-7	IL-8	IL-9
GFAP (pg/mL)	-	-	-	-	R = 0.211 *p* = 0.042	-	-	-	-	-	-	-
NF-H (pg/mL)	R = 0.232 *p* = 0.025	-	-	R = 0.226 *p* = 0.029	-	R = 0.243 *p* = 0.019	-	-	R = 0.383 *p* = 0.001	-	R = 0.310 *p* = 0.008	-
S100B (pg/mL)	-	-	-	-	-	-	R = 0.269 *p* = 0.023	R = 0.240 *p* = 0.043	R = 0.3398 *p* = 0.004	R = 0.276 *p* = 0.019	R = 0.341 *p* = 0.004	R = 0.244 *p* = 0.040
UCHL1 (pg/mL)	-	R = 0.252 *p* = 0.015	R = 0.273 *p* = 0.008	-	-	-	-	-	-	-	-	-

NF-H—neurofilament heavy chains, GFAP—glial fibrillary acidic protein, S100B—calcium-binding protein B, UCHL-1—ubiquitin C-terminal hydrolase L1, RRMS—relapsing-remitting multiple sclerosis.

**Table 4:** Presence of Gd+ lesions and the selected parameters of brain injury in the CSF in patients diagnosed de novo with RRMS.

Parameter	No Gd+ Lesions	Gd+ Lesions	*p*
*N*	69	27	
GFAP (pg/mL)	1873.84 ± 1191.32	2328.24 ± 1109.17	0.002
NF-H (pg/mL)	4.87 ± 2.28	15.01 ± 20.05	0.001
S100B (pg/mL)	11.19 ± 10.25	14.84 ± 10.86	0.061
UCHL1 (pg/mL)	128.79 ± 92.66	93.70 ± 55.65	0.633

**Table 5:** Correlations of the selected parameters of brain injury and the selected interleukins in the CSF in patients diagnosed de novo with RRMS, depending on the time from the first symptoms to diagnosis.

Parameter	IFN-γ	TNF-α	IL-1	IL-4	IL-5	IL-9	IL-10
Time from the first symptoms to diagnosis	R = 0.361 *p* = 0.000	R = 0.311 *p* = 0.003	R = 0.246 *p* = 0.019	R = 0.236 *p* = 0.025	R = 0.280 *p*= 0.007	R = 0.241 *p* = 0.22	R = 0.250 *p* = 0.017

## Data Availability

The data presented in this study are available upon request from the corresponding author. The data is not publicly available for reasons of personal data protection

## References

[1] Cotsapas C., Mitrovic M., Hafler D. (2018). Multiple sclerosis. Handb. Clin. Neurol..

[2] Lassmann H. (2018). Multiple sclerosis pathology. Cold Spring Harbor Perspectives in Medicine.

[3] Kamińska J., Koper O.M., Piechal K., Kemona H. (2017). Multiple sclerosis—Etiology and diagnostic potential. Adv. Hyg. Exp. Med..

[4] Ruiz F., Vigne S., Pot C. (2019). Resolution of inflammation during multiple sclerosis. Semin. Immunopathol..

[5] Sandi D., Fricska-Nagy Z., Bencsik K., Vécsei L. (2021). Neurodegeneration in Multiple Sclerosis: Symptoms of Silent Progression, Biomarkers and Neuroprotective Therapy—Kynurenines Are Important Players. Molecules.

[6] Stys P.K., Zamponi G.W., van Minnen J., Geurts J.J.G. (2012). Will the real multiple sclerosis please stand up?. Nat. Rev. Neurosci..

[7] Thompson A.J., Baranzini S.E., Geurts J., Hemmer B., Ciccarelli O. (2018). Multiple sclerosis. Lancet.

[8] Fischer M.T., Sharma R., Lim J.L., Haider L., Frischer J.M., Drexhage J., Mahad D., Bradl M., van Horssen J., Lassmann H. (2012). NADPH oxidase expression in active multiple sclerosis lesions in relation to oxidative tissue damage and mitochondrial injury. Brain.

[9] Lassmann H., van Horssen J., Mahad D. (2012). Progressive multiple sclerosis: Pathology and pathogenesis. Nat. Rev. Neurol..

[10] Rival M., Galoppin M., Thouvenot E. (2022). Biological Markers in Early Multiple Sclerosis: The Paved Way for Radiologically Isolated Syndrome. Front. Immunol..

[11] Khalil M., Teunissen C.E., Otto M., Piehl F., Sormani M.P., Gattringer T., Barro C., Kappos L., Comabella M., Fazekas F. (2018). Neurofilaments as biomarkers in neurological disorders. Nat. Rev. Neurol..

[12] Ferreira-Atuesta C., Reyes S., Giovanonni G., Gnanapavan S. (2021). The Evolution of Neurofilament Light Chain in Multiple Sclerosis. Front. Neurosci..

[13] Varhaug K.N., Torkildsen Ø., Myhr K.M., Vedeler C.A. (2019). Neurofilament Light Chain as a Biomarker in Multiple Sclerosis. Front. Neurol..

[14] Williams T., Zetterberg H., Chataway J. (2021). Neurofilaments in progressive multiple sclerosis: A systematic review. J. Neurol..

[15] Yang Z., Wang K.K.W. (2015). Glial fibrillary acidic protein: From intermediate filament assembly and gliosis to neurobiomarker. Trends Neurosci..

[16] Escartin C., Galea E., Lakatos A., O’Callaghan J.P., Petzold G.C., Serrano-Pozo A., Steinhäuser C., Volterra A., Carmignoto G., Agarwal A. (2021). Reactive astrocyte nomenclature, definitions, and future directions. Nat. Neurosci..

[17] Azzolini F., Gilio L., Pavone L., Iezzi E., Dolcetti E., Bruno A., Buttari F., Musella A., Mandolesi G., Guadalupi L. (2022). Neuroinflammation Is Associated with GFAP and sTREM2 Levels in Multiple Sclerosis. Biomolecules.

[18] Buffo A., Rite I., Tripathi P., Lepier A., Colak D., Horn A.P., Mori T., Götz M. (2008). Origin and progeny of reactive gliosis: A source of multipotent cells in the injured brain. Proc. Natl. Acad. Sci. USA.

[19] Kono S., Miyajima H. (2014). Aceruloplasminemia. Rosenberg’s Molecular and Genetic Basis of Neurological and Psychiatric Disease.

[20] Brenner M., Messing A. (2021). Regulation of GFAP Expression. ASN Neuro.

[21] Meier S., Willemse E.A., Schaedelin S., Oechtering J., Lorscheider J., Melie-Garcia L., Cagol A., Barakovic M., Galbusera R., Subramaniam S. (2023). Serum Glial Fibrillary Acidic Protein Compared With Neurofilament Light Chain as a Biomarker for Disease Progression in Multiple Sclerosis. JAMA Neurol..

[22] Sun M., Liu N., Xie Q., Li X., Sun J., Wang H., Wang M. (2021). A candidate biomarker of glial fibrillary acidic protein in CSF and blood in differentiating multiple sclerosis and its subtypes: A systematic review and meta-analysis. Mult. Scler. Relat. Disord..

[23] Momtazmanesh S., Shobeiri P., Saghazadeh A., Teunissen C.E., Burman J., Szalardy L., Klivenyi P., Bartos A., Fernandes A., Rezaei N. (2021). Neuronal and glial CSF biomarkers in multiple sclerosis: A systematic review and meta-analysis. Rev. Neurosci..

[24] Santos G., Barateiro A., Brites D., Fernandes A. (2020). S100B Impairs Oligodendrogenesis and Myelin Repair Following Demyelination Through RAGE Engagement. Front. Cell Neurosci..

[25] Langeh U., Singh S. (2020). Targeting S100B Protein as a Surrogate Biomarker and its Role in Various Neurological Disorders. Curr. Neuropharmacol..

[26] Camponeschi C., De Carluccio M., Amadio S., Clementi M.E., Sampaolese B., Volonté C., Tredicine M., Romano Spica V., Di Liddo R., Ria F. (2021). S100B Protein as a Therapeutic Target in Multiple Sclerosis: The S100B Inhibitor Arundic Acid Protects from Chronic Experimental Autoimmune Encephalomyelitis. Int. J. Mol. Sci..

[27] Smith K.J., Lassmann H. (2002). The role of nitric oxide in multiple sclerosis. Lancet Neurol..

[28] Li R., Wang J., Xie W., Liu J., Wang C. (2020). UCHL1 from serum and CSF is a candidate biomarker for amyotrophic lateral sclerosis. Ann. Clin. Transl. Neurol..

[29] Sjölin K., Kultima K., Larsson A., Freyhult E., Zjukovskaja C., Alkass K., Burman J. (2022). Distribution of five clinically important neuroglial proteins in the human brain. Mol. Brain.

[30] Brophy G.M., Mondello S., Papa L., Robicsek S.A., Gabrielli A., Tepas J., Buki A., Robertson C., Tortella F.C., Hayes R.L. (2011). Biokinetic Analysis of Ubiquitin C-Terminal Hydrolase-L1 (UCH-L1) in Severe Traumatic Brain Injury Patient Biofluids. J. Neurotrauma.

[31] Papa L., Akinyi L., Liu M.C., Pineda J.A., Tepas J.J., Oli M.W., Zheng W., Robinson G., Robicsek S.A., Gabrielli A. (2010). Ubiquitin C-terminal hydrolase is a novel biomarker in humans for severe traumatic brain injury. Crit. Care Med..

[32] Górska E., Tylicka M., Hermanowicz A., Matuszczak E., Sankiewicz A., Gorodkiewicz E., Hermanowicz J., Karpińska E., Socha K., Kochanowicz J. (2023). UCHL1, besides leptin and fibronectin, also could be a sensitive marker of the relapsing–remitting type of multiple sclerosis. Sci. Rep..

[33] Lu H., Wu P.F., Zhang W., Liao X. (2021). Circulating Interleukins and Risk of Multiple Sclerosis: A Mendelian Randomization Study. Front. Immunol..

[34] Silva A.C., Lobo J.M.S. (2019). Cytokines and Growth Factors.

[35] Ryff J.C., Pestka S. (2013). Interferons and Interleukins. Pharmaceutical Biotechnology.

[36] Morawiec N., Techmański T., Tracz K., Kluska A., Arendarczyk M., Baran M., Adamczyk B., Czuba Z., Bronikowska J., Adamczyk-Sowa M. (2023). The comparative analysis of selected interleukins and proinflammatory factors in CSF among de novo diagnosed patients with RRMS. Clin. Neurol Neurosurg..

[37] Thompson A.J., Banwell B.L., Barkhof F., Carroll W.M., Coetzee T., Comi G., Correale J., Fazekas F., Filippi M., Freedman M.S. (2018). Diagnosis of multiple sclerosis: 2017 revisions of the McDonald criteria. Lancet Neurol..

[38] Houser B. (2012). Bio-Rad’s Bio-Plex® suspension array system, xMAP technology overview. Arch. Physiol. Biochem..

[39] Herrera M.I., Kölliker-Frers R.A., Otero-Losada M., Perez Lloret S., Filippo M., Tau J., Capani F., Villa A.M. (2019). A Pilot Cross-Sectional Study to Investigate the Biomarker Potential of Phosphorylated Neurofilament-H and Immune Mediators of Disability in Patients With 5 Year Relapsing-Remitting Multiple Sclerosis. Front. Neurol..

[40] Shehab A.A., Solima D.A., Abdel-Hafeez M.A., Mohamed S.M. (2019). Serum Phosphorylated Neurofilament Heavy Chain Level in Relapsing Remitting Multiple Sclerosis in Correlation to Disease Activity and Disability. Egypt J. Immunol..

[41] Tüzün E., Şanlı E.Ş., Akbayır E., Türkoğlu R. (2021). Phosphorylated neurofilament heavy chain (pNFH) in clinically isolated syndrome and multiple sclerosis. Arch. Neuropsychiatry.

[42] Martín-Aguilar L., Presas-Rodriguez S., Rovira À., Capellades J., Massuet-Vilamajó A., Ramió-Torrentà L., Tintoré M., Brieva-Ruiz L., Moral E., Cano-Orgaz A. (2022). Gadolinium-enhanced brain lesions in multiple sclerosis relapse. Neurologia (Engl. Ed.).

[43] Matsushige T., Ichiyama T., Anlar B., Tohyama J., Nomura K., Yamashita Y., Furukawa S. (2008). CSF neurofilament and soluble TNF receptor 1 levels in subacute sclerosing panencephalitis. J. Neuroimmunol..

[44] Daoud H., Alharfi I., Alhelali I., Charyk Stewart T., Qasem H., Fraser D.D. (2014). Brain Injury Biomarkers as Outcome Predictors in Pediatric Severe Traumatic Brain Injury. Neurocrit. Care.

[45] Lund B.T., Ashikian N., Ta H.Q., Chakryan Y., Manoukian K., Groshen S., Gilmore W., Cheema G.S., Stohl W., Burnett M.E. (2004). Increased CXCL8 (IL-8) expression in Multiple Sclerosis. J. Neuroimmunol..

[46] Rossi S., Motta C., Studer V., Macchiarulo G., Germani G., Finardi A., Furlan R., Martino G., Centonze D. (2015). Subclinical central inflammation is risk for RIS and CIS conversion to MS. Mult. Scler. J..

[47] Stampanoni Bassi M., Iezzi E., Landi D., Monteleone F., Gilio L., Simonelli I., Musella A., Mandolesi G., De Vito F., Furlan R. (2018). Delayed treatment of MS is associated with high CSF levels of IL-6 and IL-8 and worse future disease course. J. Neurol..

[48] Kassubek R., Gorges M., Schocke M., Hagenston V.A., Huss A., Ludolph A.C., Kassubek J., Tumani H. (2017). GFAP in early multiple sclerosis: A biomarker for inflammation. Neurosci. Lett..

[49] Ayrignac X., Le Bars E., Duflos C., Hirtz C., Maleska Maceski A., Carra-Dallière C., Charif M., Pinna F., Prin P., Menjot de Champfleur N. (2020). Serum GFAP in multiple sclerosis: Correlation with disease type and MRI markers of disease severity. Sci. Rep..

[50] Di Sante G., Amadio S., Sampaolese B., Clementi M.E., Valentini M., Volonté C., Casalbore P., Ria F., Michetti F. (2020). The S100B Inhibitor Pentamidine Ameliorates Clinical Score and Neuropathology of Relapsing—Remitting Multiple Sclerosis Mouse Model. Cells.

[51] Michetti F., D’Ambrosi N., Toesca A., Puglisi M.A., Serrano A., Marchese E., Corvino V., Geloso M.C. (2019). The S100B story: From biomarker to active factor in neural injury. J. Neurochem..

[52] Bartosik-Psujek H., Psujek M., Jaworski J., Stelmasiak Z. (2011). Total tau and S100b proteins in different types of multiple sclerosis and during immunosuppressive treatment with mitoxantrone. Acta Neurol. Scand..

[53] Rejdak K., Petzold A., Kocki T., Kurzepa J., Grieb P., Turski W.A., Stelmasiak Z. (2007). Astrocytic activation in relation to inflammatory markers during clinical exacerbation of relapsing-remitting multiple sclerosis. J. Neural Transm..

[54] Barateiro A., Afonso V., Santos G., Cerqueira J.J., Brites D., van Horssen J., Fernandes A. (2016). S100B as a Potential Biomarker and Therapeutic Target in Multiple Sclerosis. Mol. Neurobiol..

[55] Bianchi R., Giambanco I., Donato R. (2010). S100B/RAGE-dependent activation of microglia via NF-κB and AP-1. Neurobiol. Aging.

[56] Xu J., Wang H., Won S.J., Basu J., Kapfhamer D., Swanson R.A. (2016). Microglial activation induced by the alarmin S100B is regulated by poly(ADP-ribose) polymerase-1. Glia.

[57] Gunawan P.I., Saharso D., Sari D.P. (2019). Correlation of serum S100B levels with brain magnetic resonance imaging abnormalities in children with status epilepticus. Korean J. Pediatr..

[58] Mi Z., Graham S.H. (2023). Role of UCHL1 in the pathogenesis of neurodegenerative diseases and brain injury. Ageing Res. Rev..

[59] Dougan M., Dranoff G., Dougan S.K. (2019). GM-CSF, IL-3, and IL-5 Family of Cytokines: Regulators of Inflammation. Immunity.

[60] Wang K., Song F., Fernandez-Escobar A., Luo G., Wang J.H., Sun Y. (2018). The Properties of Cytokines in Multiple Sclerosis: Pros and Cons. Am. J. Med. Sci..

[61] Mathur D., Mishra B.K., Rout S., Lopez-Iranzo F.J., Lopez-Rodas G., Vallamkondu J., Kandimalla R., Casanova B. (2021). Potential Biomarkers Associated with Multiple Sclerosis Pathology. Int. J. Mol. Sci..

[62] Yang J., Hamade M., Wu Q., Wang Q., Axtell R., Giri S., Mao-Draayer Y. (2022). Current and Future Biomarkers in Multiple Sclerosis. Int. J. Mol. Sci..

